# Economic Optimal Allocation of Mine Water Based on Two-Stage Adaptive Genetic Algorithm and Particle Swarm Optimization

**DOI:** 10.3390/s22030883

**Published:** 2022-01-24

**Authors:** Zihang Zhang, Yang Liu, Lei Bo, Yuangan Yue, Yiying Wang

**Affiliations:** 1 School of Mechanical Electronic & Information Engineering, China University of Mining and Technology (Beijing), Beijing 100083, China; zhangzihang709@163.com (Z.Z.); liuyangebox@126.com (Y.L.); 18811028514@163.com (Y.Y.); 2 School of Mechanical and Equipment Engineering, Hebei University of Engineering, Handan 056038, China; yatesyy@163.com

**Keywords:** optimal allocation, economic reuse, GAPSO hybrid algorithm, two-stage optimization, adaptive adjustment

## Abstract

The waste mine water is produced in the process of coal mining, which is the main cause of mine flood and environmental pollution. Therefore, economic treatment and efficient reuse of mine water is one of the main research directions in the mining area at present. It is an urgent problem to use an intelligent algorithm to realize optimal allocation and economic reuse of mine water. In order to solve this problem, this paper first designs a reuse mathematical model according to the mine water treatment system, which includes the mine water reuse rate, the reuse cost at different stages and the operational efficiency of the whole mine water treatment system. Then, a hybrid optimization algorithm, GAPSO, was proposed by combining genetic algorithm (GA) and particle swarm optimization (PSO), and adaptive improvement (TSA-GAPSO) was carried out for the two optimization stages. Finally, simulation analysis and actual data detection of the mine water reuse model are carried out by using four algorithms, respectively. The results show that the hybrid improved algorithm has better convergence speed and precision in solving the mine water scheduling problem. TSA-GAPSO algorithm has the best effect and is superior to the other three algorithms. The cost of mine water reuse is reduced by 9.09%, and the treatment efficiency of the whole system is improved by 5.81%, which proves the practicability and superiority of the algorithm.

## 1. Introduction

Since the concept of green mine was put forward, the mineral industry has responded positively [1,2,3]. Under the strict requirements of carbon emission control, how to minimize environmental pollution under the premise of mining has become the current goal of the mineral industry [4,5]. Mine water, as a derivative in the process of mining, is also a resource containing pollutants [6,7]. Mine water cannot be reused directly; if discharged directly, it will cause irreversible damage to the surrounding environment of the mining area and seriously affect the life of the mining area and endanger the safety of the mine [8,9]. Therefore, how to meet the need for mineral exploitation as well as achieve mine water efficient treatment is an important research direction at present. The researchers designed and developed a set of automatic heavy metal filtration devices for environmental pollution caused by heavy metals in mining wastewater, which achieved excellent treatment effect and reduced treatment cost [10]. According to the characteristics of coal mine water quality, the researchers proposed an improvement scheme for the original underground water silo, and the results proved that the scheme improved the underground reuse efficiency of mine water and reduced the reuse cost [11]. The author proposed a neutralization scheme and treatment technology for the environmental pollution of acid mine water, and the results proved that the scheme had a good treatment effect [12]. The efficient utilization of mine water not only stays in the state of water quality treatment but also has an important influence on reuse. The study of the above literature is mainly aimed at the water quality treatment and flow of mine water. The efficient utilization of mine water not only stays in the stage of water quality treatment but also has an important impact on reuse. At present, there are very few studies on mine water scheduling, and most coal mines use the method of nearby reuse or unified reuse. Therefore, this paper studies the reuse mechanism of mine water.

With the development of information technology and intelligent technology, more and more excellent algorithms have been invented. As one of the intelligent algorithms, the optimal scheduling algorithm has been in the state of development in recent years. There have been the particle swarm optimization algorithm [13], slime mold algorithm [14], whale feeding algorithm [15], Harris Eagle algorithm [16], Runge Kutta optimization algorithm [17] and so on. These algorithms have been well used in life, for example, the movement track of underground scraper [18], the parameter selection of solar photovoltaic panel [19], the complementary charging system of photovoltaic power grid [20], the job shop scheduling [21] and the nonlinear thermodynamic buckling of intelligent sandwich panel [22]. Particle swarm optimization algorithm and genetic algorithm, as the most commonly used optimization algorithms, have also been applied to all walks of life. In order to eliminate the problem of EEG single self-interference, the author optimized and tested five PSO algorithms and finally proved that the improved versions of NLI and LDI were most suitable for filtering ANC [23]. The researchers developed a method to predict a crystal structure through PSO algorithm and verified that it has a high success rate [24], indicating the technical prospect of PSO algorithm in crystal prediction. Some researchers proposed a PSO-based support vector machine parameter determination and parameter selection method [25] and verified that this method has high value for support vector machine parameter determination and selection. The researchers proposed a clustering analysis method based on genetic algorithm (GA) and verified its feasibility and superiority [26]. A single algorithm improvement and optimization sometimes cannot meet the needs of real, so many researchers improve the algorithm. Ratnaweera introduced time-varying acceleration coefficient and time-varying inertia factor in the process of particle swarm optimization to control the convergence accuracy of the algorithm under different iterations [27]. The author uses the different components of the population co-optimization vector solution to control the direction of particle optimization [28]. Some authors classified the evolution states of PSO and adopted different convergence optimization strategies in different states [29] to improve the convergence speed, accuracy and reliability of the algorithm. Some researchers hybridized the particle swarm with adaptive inertial weight and the chaotic particle population to form an adaptive search algorithm with chaotic search capability [30]. In order to introduce fixed point theory into the algorithm and transform the optimal problem into fixed point problem, some researchers proposed an improved genetic algorithm based on J1 triangulation [31]. Some researchers have also made optimization improvements in crossover and mutation probability [32,33]. A single optimization algorithm sometimes cannot meet the actual needs, so some researchers propose a hybrid algorithm of particle swarm optimization and genetic algorithm and apply the optimization results to recursive neural network analysis [34], but the speed and precision of convergence need to be improved. The author took advantage of the optimal sharing characteristics of PSO to guide the inheritance of GA and proposed a hybrid evolutionary clustering algorithm based on PSO algorithm and GA algorithm to reduce the setup time of surface mounting technology [35]. Simulation comparison shows that this method has certain advantages in reducing machine production time and idle time. In addition, some researchers proposed a hybrid GAPSO algorithm based on GA and PSO to improve the accuracy of the scheduling strategy of FMS and improve the global optimization capability of PSO by using the crossover and variation characteristics of GA [36] and verified the superiority of the hybrid algorithm in improving operation efficiency. For the hybrid improvement of particle swarm optimization and genetic algorithm, researchers applied the particle swarm optimization algorithm to the crossover operator [37] and mutation operator [38] of genetic algorithm to improve the optimization ability of genetic algorithm. For the hybrid algorithm, some researchers analyzed its optimization mechanism and enhanced the ability of adaptive sampling and local in search, which provided certain guidance for the study of this paper [39].

Based on the above research results, it can be found that the optimal scheduling algorithm is widely used. A single particle swarm optimization algorithm and a single genetic algorithm can solve some optimization problems, but with the development of the algorithm, it is found that the optimization effect of the hybrid algorithm is sometimes better than that of the single algorithm. A new hybrid mechanism is proposed to solve the problem of convergence speed and precision of single algorithm. The hybrid scheme of the two algorithms proposed in this paper has not been applied in mine water scheduling, so it is worth testing and studying. Therefore, this paper applies the sharing mechanism and strong local search ability of PSO to the genetic iteration of a genetic algorithm to find the optimal scheduling scheme of mine water. The main research contents of this paper are as follows:

1. According to the scheduling status of mine water, analyze the demand for water in a mining area and construct the objective function of economic reuse.
2. The characteristics of particle swarm optimization and genetic algorithm are analyzed. In addition, carry on the fusion improvement according to their characteristics. The results show that the hybrid algorithm has better convergence effect than the original algorithm.
3. Make adaptive improvement on the two stages of the hybrid algorithm, so that the hybrid algorithm can be further optimized. The comparison results show that the improved hybrid algorithm is better than the hybrid algorithm in convergence speed and accuracy.
4. Use four algorithms to simulate the reuse model of mine water, and then compare it with the actual production scheduling situation of mine water under the nearby principle. Simulation results show that mine water scheduling based on this algorithm has better economy and efficiency compared with the nearby principle.

The structure of this paper is as follows. The Section 2 describes the state of mine water treatment and reuse and establishes the corresponding mathematical model. The Section 3 is the theoretical description of the algorithm, hybrid improvement and adaptive adjustment. The Section 4 is for the actual water used in the sea coal mine simulation test. The Section 5 in the work of this paper.

## 2. Optimal Scheduling Model

In order to understand the present situation of mine water reuse in detail, this paper investigates the current situation of water inrush and water use in Dahaize Coal mine. Through the analysis of its treatment system and reuse process, the water demand model of the mining area is constructed, and the objective function is set up.

### 2.1. Mining Demand Models

**Figure 1:**
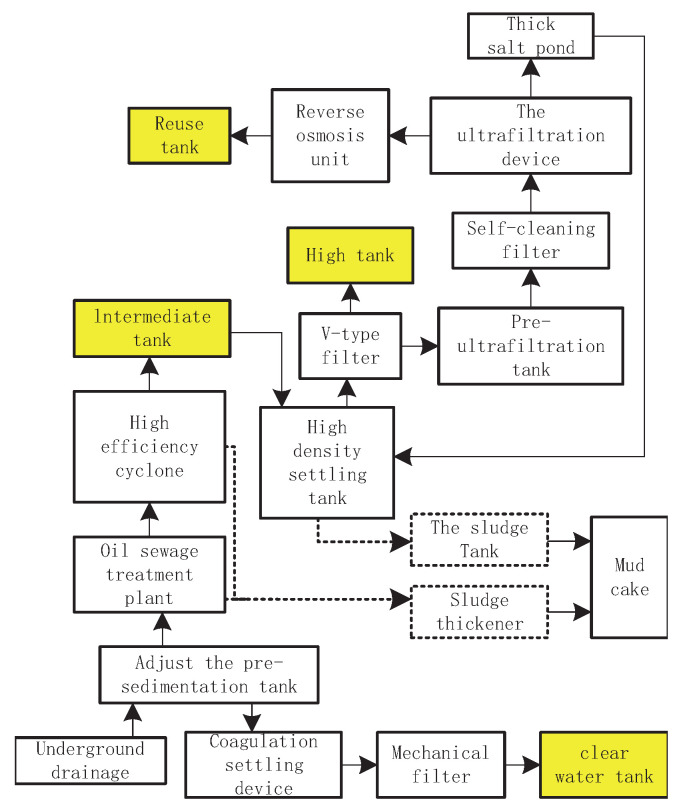
Flow chart of mine water treatment process.

Firstly, the mine water reuse system is investigated and analyzed. Mine water pretreatment station carried out conventional treatment and hard treatment for all underground drainage. Some underground drainage was deeply treated and then used for coal mine production and living water, and the rest was sent to the mine water treatment plant for in-depth treatment. The conventional treatment unit consists of four parts: dehardening, coagulation and sedimentation, filtering and disinfection. The advanced treatment process includes self-cleaning filtration, ultrafiltration, reverse osmosis and disinfection process. After the above treatment, the mine water that meets the requirements can be supplied to the water point of the mining area, and the rest can be transported to the water treatment plant or power plant around the coal mine. In Figure 1, the regulated pre-settling pool, clear pool, middle pool, high pool and reuse pool are responsible for water quality monitoring and allocation. Among them, the regulated pre-settling pool is at the beginning of mine water gushing into the system, so the water quality among them is only tested, not allocated. The specific mine water treatment process is shown in Figure 1:

Mining area water is mainly divided into two parts, namely underground coal mine water and ground water. This paper classifies water points according to water conditions in mining areas and defines the following five equations. The specific content mainly includes the following aspects:

Safe water for underground production in coal mines. Production water includes hydraulic support water, grouting water and cooling water, underground safe water includes underground fire control water and underground dust removal water, and its demand is as Equation (1):

(1) $$Q_{1}=S\times n+G\times \alpha +Y_{i}\times m+\left(L_{j}+C_{k}\right)\times t$$

In the formula, *S* represents the average water consumption of underground fire; *n* is the number of underground fires; *G* represents the volume of grouting; α stands for water proportion, usually 0.6; *Y* represents the water consumption of hydraulic support; *m* represents the number of hydraulic supports; $L_{j}\;$ represents the average cooling water consumption; $C_{k}$ represents the average water consumption of underground dust removal equipment; *t* represents time, in hours.

Mining ground safety protection water $Q_{2}$. Safe water mainly includes ground fire fighting and dust removal. Compared with underground fire fighting and dust removal, ground fire fighting water consumption is less, and the storage water supply pool is relatively small. For dust removal water, the frequency of road sprinkling is lower than that of the underground roadway. Therefore, the safe water demand on the ground is as Equation (2):

(2) $$Q_{2}=q\times \left(D\times d\right)/365+S\times n$$

In the formula, *q* represents sprinkling water quota; *D* is the area of the road; *d* represents the average number of sprinkler days per year; *S* represents the average water consumption of ground fire; *n* is the average number of ground fires per year.

Coal mine processing water $Q_{3}$. Ground production water refers to the secondary processing of mined coal and the treatment of coal slime, mainly including coal preparation water, heat exchange station water, cooling water and boiler water. Its forecast demand is as Equation (3):

(3) $$Q_{3}=X_{Z}\times T_{1}+X_{T}\times T_{2}+H\times T_{3}+G_{i}\times T_{4}+L_{j}\times T_{5}$$

In the formula, $X_{Z}$ and $X_{T}$ represent the average water consumption of the heavy medium process and jigging process in the coal preparation plant, respectively; *T* represents the average evaporation and water loss of the heat exchange station; *G* represents water consumed in the coal water slurry boiler system; *L* represents the average water consumption of cooling equipment; $T_{i}$ indicates the running time of the corresponding device.

Mining area residents living water $Q_{4}$. In order to ensure the quality of life in the mining area, the residential water has higher quality requirements. The predicted demand for greening and drinking in the main mining areas is as Equation (4):

(4) $$Q_{4}=N\times g+S\times l$$

In the formula, *N* represents the total number of residents in the mining area, *g* represents the water consumption standard per capita in the mining area, *S* represents the green area in the mining area and *l* represents the average water consumption for greening.

The problem of mine water dispatching and reuse can be described as follows. In the process of mine water treatment and reuse, mine water is constantly pouring into the treatment system, and the system is in an uninterrupted running state. The utilization of underground and ground water resources in coal mine is in a random state, and the reuse scheduling system arranges the scheduling scheme reasonably according to the demand of underground and ground water and transfers the water in the treatment process to the water point in the mining area. In mine water reuse, the reuse rate η is determined by measuring the reuse amount of each reuse point in the mine. The calculation formula is as Equation (5):

(5) $$\eta =\sum_{i=1}^{M}\frac{Q_{i}}{S}$$

In the formula, *M* represents the reuse point of the mining area, $Q_{i}$ represents the recycling amount of the *i* reuse point of the mining area and *S* represents the water inflow of the mining area.

### 2.2. Objective Functions of Economic Reuse in Mining Area

Complex processing technology and high cost of treatment are the reasons for the shortage of mine water treatment. Therefore, the reasonable dispatch and distribution of mine water is one of the problems that needs to be solved at present. The research goal of this paper is to calculate a reasonable solution based on the demand analysis of mine water, combined with the constraints of water quality and quantity of each water point in the mining area, so as to achieve the highest recycling rate of mine water and the lowest treatment cost on the basis of meeting the production and life of the mining area. In this paper, the reciprocal of mine water consumption cost and the sum of reuse efficiency are taken as the objective function, and the water quality and quantity of each water point are taken as the constraint conditions. The operation cost of mine water dispatching is mainly electricity cost generated by pump work, and the treatment and purification cost includes pharmaceutical treatment cost and labor and mechanical cost generated by purification process. Establish a mathematical model of system optimal scheduling for a certain period of time *T* as Equation (6).

(6) $$\min fit=\sum_{i=1}^{M}\sum_{j=1}^{N}\left(g_{1}\frac{\left(Y_{ij}+E_{ij}+\delta \right)\times Q_{ij}}{Z\times S}+g_{2}\frac{Q_{ij}\times T}{t_{i}\times Z}\right)$$

In the formula, $S_{i}$ represents the maximum reconsumption of mine water; *i* represents the stage of mine water treatment; *j* represents the classification of water points; $Y_{i}$ represents the cost of chemical drugs; $E_{i}$ represents the electricity cost of mine water treatment; $q_{i}$ represents the water consumption of *j* water points in stage *i*; δ represents the equipment repair cost after the water treatment is converted into 0.16 RMB/ton; *Z* represents the treatment cost in the final stage; $g_{1}$ and $g_{2}$ represent the weight coefficients, 0.6 and 0.4, respectively; $t_{i}$ represents unit reuse time of mine water in each stage; and *T* represents the maximum treatment time.

### 2.3. The Constraint

Water quality condition in a mining area. As mine water mostly belongs to acidic liquid containing a variety of minerals, it needs to go through a variety of reactions before it can be used. Therefore, each level of mine water treatment has a lower limit and upper limit of water quality as Equation (7), and the water quality standard of each mine water point can only be invoked within this range. According to the characteristics of water quality and the demand of reuse, the following three inequalities are formulated for the reuse system.

(7) $$G_{i\min}<G_{i}<G_{i\max}$$

Here, $G_{i}$ represents the water quality requirement standard of water point, $G_{imin}$ represents the minimum water quality standard of mine water in stage *i* and $G_{imax}$ represents the highest water quality standard that can be achieved in stage *i*.

Mine water inflow water balance. In any time period of system operation, the water inflow of the mine should be equal to the sum of water consumption and the displacement of the mine as Equation (8). In the process of treatment and purification, water in sludge and water consumed by evaporation will inevitably appear, so these are ignored in this paper.

(8) $$W=\sum_{i=1}^{M}Q_{i}+D$$

Here, *W* represents the water inflow of the mine, $Q_{i}$ represents water consumption of each mining area and *D* represents the water discharged from the system after completion of treatment.

Mine water treatment speed. Although the mine water treatment is in continuous operation, the water in the recycling pool in actual production is not infinite but needs to be stored for a certain period of time, especially for the process of dosing precipitation, which needs a long period of time. The speed limit is shown in Equation (9).

(9) $$V_{i\min}<V_{i}<V_{i\max}$$

In the formula, $V_{i}$ represents the mine water purification speed in the i treatment stage, $V_{imax}$ indicates the highest processing speed in phase *i* and $V_{imin}$ indicates the lowest processing speed in phase *i*.

Based on the above conclusions, four constraint conditions of mine water optimal scheduling model are obtained in this paper, including one equality constraint condition and three nonlinear constraint conditions. So the optimal scheduling model proposed in this paper is an optimization problem with nonlinear constraints. For this kind of problem, the penalty function is introduced to transform the optimal scheduling problem into an unconstrained optimization problem and then solve it.

### 2.4. Penalty Functions

Penalty function has a great advantage in solving optimization problems under various constraints, because it can replace a constrained problem with an identical unconstrained problem [40]. Therefore, the constraint conditions in the process of mine water treatment are converted to the calculation of penalty functions, and the calculation of penalty values can refer to Equation (10) [41]:

(10) $$\varphi =\sum_{n=1}^{N}{\left(\max\left\{0,-u_{n}\left(\vec{x}\right)\right\}\right)}^{2}+\sum_{h=1}^{H}\left({\left|z_{h}\left(\vec{x}\right)\right|}^{2}\right)$$

For Formula (10), φ represents the penalty value, *N* represents the number of inequality constraints in the optimal scheduling problem, *H* represents the number of equality constraints, $u_{n}\left(\vec{x}\right)$ represents the result of the transformation of the *N*th inequality constraint and $z_{h}\left(\vec{x}\right)$ represents the result of the transformation of h equality constraints.

If the variable is beyond the inequality constraint condition of the given limits, the penalty value is $|-u_{n}\left(\vec{x}\right){|}^{2}$; otherwise, it is 0. If a variable is beyond the equality constraint conditions of the given limits, the penalty value is ${|z_{h}\left(\vec{x}\right)|}^{2}$; otherwise, it is 0. A fairly large positive integer δ can then be multiplied by the penalty value [42] and appended to the end of the objective function proposed in this paper. Since the optimal objective function in this paper is to find its minimum value, it can be added with penalty value to form an augmented function relative to the original objective function as Equation (11):

(11) fin(fi)=f+δφ

## 3. Hybrid Improved Algorithm Based on Genetic Algorithms and Particle Swarm Optimization

This paper is devoted to solving the optimization scheduling problem of mine water, mainly including one to many and many to many mine water reuse methods. In this paper, the mine water reuse system in the Dahaize mining area is investigated, as described in Section 2.1. The mine water reuse problem in this paper is summed up as a multi-objective optimization problem. Both particle swarm optimization (PSO) and genetic algorithm (GA) have great advantages and convenience for solving multi-objective optimization problems [43,44,45,46]. Therefore, this paper will make use of the respective advantages of particle swarm optimization and genetic algorithm to optimize the mine water distribution.

The stability of the optimization algorithm is affected by the emphasis among the core algorithms. The algorithm compares and selects all individuals in the population with the optimal individuals in the previous generation, so as to obtain the evolutionary direction of the next generation of individuals [47,48]. It can be seen that the PSO algorithm focuses more on the overall optimization ability. In the genetic algorithm, the algorithm cross-operates several excellent individuals of the first generation through selection operation [49]. This process reflects the characteristics of the gene transmission of excellent individuals from generation to generation and also reflects the information exchange between individuals. It can be seen that the genetic algorithm lays more emphasis on the local search ability of the algorithm. Therefore, the fusion and balance of the two algorithms can better solve the problem of optimal scheduling.

### 3.1. Overview of PSO

Particles in the particle swarm optimization algorithm are considered as massless and volumeless particles when searching for spatial motion [50,51,52]. Suppose there are n mass less and fewer particles in the D-dimensional search space. In swarm space, every particle has two properties; they are the current position of the particle and the speed at which it travels [53]. The position of the No.*i* particle is represented by *X_id_*, expressed as $X_{id}=\left[x_{i1},x_{i2},x_{i3}\ldots x_{iD}\right]$, (d=1,2,3…D). The flight speed and direction of the *i*th particle are represented by *V_id_*, expressed as $V_{id}=\left[v_{i1},v_{i2},v_{i3}\ldots v_{iD}\right],\left(d=1,2,3\ldots D\right)$. $X_{i}$ was substituted into the fitness function to judge the quality of the current population by the size of fitness value. In order to guide the direction and speed of the next population movement, particle swarm optimization has two special mechanisms, namely the individual extreme value and the population extreme value [54]. The former represents the best position searched by the particle, namely *Pbest*_*id*_ = (*p*_1*d*_, *p*_2*d*_, *p*_3*d*_, …, p_*nd*_), *Gbest*_*gd*_ = (*G*_1*d*_, *G*_2*d*_, *G*_3*d*_, …, *G*_*gd*_).

The iteration formula of each update in the algorithm iteration process as Equations (12) and (13) [50]:

(12) $$V_{id}^{k+1}=\omega V_{id}^{k+1}+C1\varepsilon \left(P_{best\;}^{k}-x_{id}^{k}\right)+C2\mu \left(G_{best\;}^{k}-x_{id}^{k}\right)$$

(13) $$X_{id}^{k+1}=X_{id}^{k}+V_{id}^{k+1}\ldots d=1,\;2,\;3\ldots D$$

In the above formula, Vid represents the speed and direction of the ith particle in the D-dimensional space. $X_{id}$ is the position of the ith particle in *D*-dimensional space. *C*1 and *C*2 represent the weight of the optimal value of particle and population in the historical search process, respectively. It is always going to be 2. The parameters ε and μ are random numbers distributed over the interval [55]. Pbest and Gbest represent the individual extreme value and global extreme value of particle population, respectively. $P_{best}^{k}-x_{id}^{k}$ is called self-awareness, $G_{best}^{k}-x_{id}^{k}$ is called the social cognitive [55]. Its expression is as Equations (14) and (15):

(14) $$Pbest{}_{i}\left(k\right)=\mathrm{argmin}\left\{fit\left(X_{i}\left(1\right)\right),fit\left(X_{i}\left(2\right)\right),fit\left(X_{i}\left(3\right)\right),\ldots ,fit\left(X_{i}\left(k\right)\right)\right\}$$

(15) $$Gbest\left(k\right)=\mathrm{argmin}\left\{{Pbest}_{1}\left(k\right),Pbest{}_{2}\left(k\right),Pbest{}_{3}\left(k\right),\ldots ,Pbest{}_{4}\left(k\right)\right\}$$

Omega represents the particle weight coefficient [56], which is usually a decreasing linear change parameter, specifically defined as Equation (16).

(16) $$\omega_{k}=\omega_{\max}-\left(\frac{\omega_{\max}-\omega_{\min}}{K_{\max}}k\right)$$

$\omega_{\max}$ = 0.9, $\omega_{\min}$ = 0.4, $K_{\max}$ is the maximum number of iterations, *k* is the current number of iterations. It can be seen that with the continuous iteration, the state of the population is constantly changing.

### 3.2. An Overview of Genetic Algorithms

The basic principle of the genetic algorithm is derived from the long and long evolutionary process of species from simple and low to complex and high [57]. In the search process, the genetic algorithm should not only consider the basic information of the current population but also consider the population characteristics formed by the accumulation of previous iterative experience and adopt the unique process of inheritance, crossover and mutation to control the search results to move to the global optimal solution [58]. The solution process is shown as follows:

(1) Code design

In the process of solving the genetic algorithm, the practical problem is transformed into the genetic algorithm chromosome structure, that is, the genetic algorithm coding. The encoding mode determines the optimization performance and efficiency of the algorithm to a certain extent, and the encoding mode mainly includes binary, ordered string encoding and real number encoding. The fitness function selected in this paper is continuous function with small variation range, so the binary coding method with a simple coding method and easy crossover and mutation is selected [59].

(2) Generating initial population

Before the basic genetic operation, an initial population consisting of several initial feasible solutions needs to be constructed. The most important measure of the initial population is population size *n*. Under the condition that constraint conditions are met, the total amount of *n* individuals is *Q*, that is, the water demand of the mining area. The larger the population size n is, the more individual species are selected, crossed and mutated, which is helpful to avoid falling into the local optimal solution and improve the probability of finding the global optimal solution.

(3) Fitness function

The design of fitness function is to evaluate the adaptability of individuals, which plays a decisive role in the optimization of genetic algorithm and is the driving force of algorithm evolution [60]. The selection of fitness function is very important in the operation of the genetic algorithm. It will affect the convergence efficiency and the ability of the optimal solution. This paper designs the fitness function on the basis of the objective function, and the formula is as Equation (17):

(17) $$GAf_{s}=f-\min f+e$$

In the formula, $GAf_{s}$ represents the fitness function, min*f* represents the minimum value of the objective function, e represents a smaller number and a smaller number is added to make the fitness function greater than 0. In this paper, $10^{-6}$ is taken.

(4) Choose

The selection operation of genetic algorithm is to retain the excellent genes in the population, so that the individual genes with high fitness value can be better inherited to the next generation, so as to improve the convergence speed and accuracy of the algorithm in the operation process, so as to achieve better calculation results. In this paper, roulette selection method is selected, that is, the proportion of individual fitness value in the whole population, where the proportion calculation formula is Equation (18):

(18) $$P_{i}=\frac{GAf(i)}{\sum_{1}^{n}GAf\left(i\right)}$$

This method cannot guarantee that individuals with high fitness value will be selected. If chromosomes with low fitness value are selected, it is likely to cause population degradation and decrease the convergence rate.

(5) Crossover and variation

In the running process of genetic algorithm, two individuals of the first generation exchange information according to certain methods and produce two offspring individuals [61]. Firstly, a certain crossover probability is set, and then the location of the crossover point in the gene is generated according to the crossover probability. The genes behind the crossover point are the genes exchanged between the first two individuals, resulting in the second generation of individuals. The crossover operation is described in binary terms, with chromosomes $X_{1}$, $X_{2}$. First, set a crossover mutation probability $C_{rate}$, which is 0.8 in this paper, and generate a random decimal $c_{rate}$ as Equation (19).

(19) $$c_{rate\;}<C_{rate\;}$$

The following crossover operation occurs.

**Figure 2:**
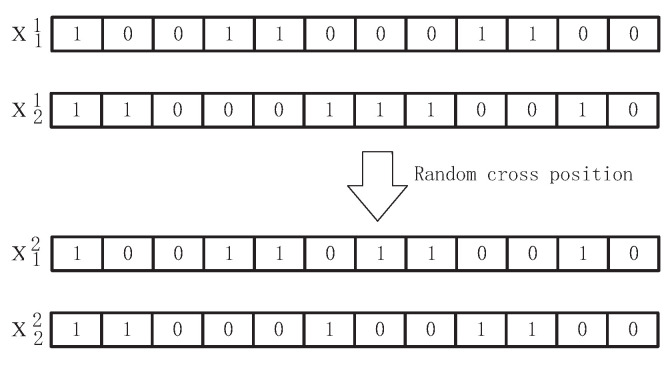
Schematic diagram of binary encoding crossing.

Mutation operation and crossover operation are used to increase the diversity of the genetic algorithm in the process of optimization. The interactions are shown in Figure 2. The main purpose is to improve the local search ability of genetic algorithm and mutation is to change a gene of the parent with a certain probability, while maintaining the logical relationship of genes, compilation operation is as Equation (20): First, set a mutation probability $M_{rate}$, this paper takes 0.8, randomly generate a decimal $m_{rate}$, when:

(20) $$m_{rate\;}<M_{rate\;}$$

The following mutation operation occurs.

**Figure 3:**
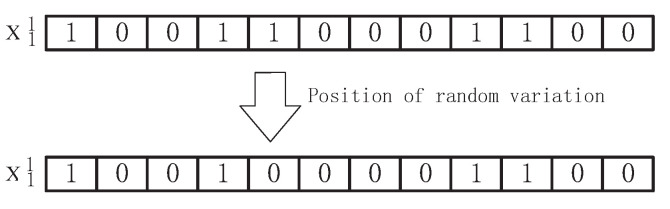
Schematic diagram of binary code variation.

For the setting of crossover and genetic probability parameters, different parameter selection has different effects on the quality of the algorithm, but the setting of parameters at present mainly depends on the experience of predecessors. The mutation process is shown in Figure 3.

### 3.3. Overview of the GAPSO

In this paper, a hybrid GAPSO algorithm is proposed to guide the evolution direction of genetic algorithm particles by using the extreme value sharing characteristic of the particle swarm optimization algorithm. GAPSO hybrid algorithm is based on the framework of genetic algorithm, which ensures the evolution of population through individual selection, crossover and mutation operation within the genetic algorithm. At the same time, particle swarm optimization algorithm is used as an assistant to assist the evolution of all individuals to move toward the extreme point. In the genetic algorithm, the operation of chromosomes makes the whole evolve in the optimal direction through the selection and crossover between individuals, and it is inevitable that individual degeneration will occur, which will slow down the rhythm of the whole and affect the judgment of the algorithm as a whole. Therefore, in the evolutionary process of genetic algorithm, fitness value (same as individual extreme value) is discriminated for all individuals. For degenerate particles, particle swarm optimization algorithm is used to give them the evolutionary direction and search for optimization again, so that all individuals have the same evolutionary direction.

The solving steps of the GAPSO algorithm are as follows:

Step 1: Initialize the population size, iteration times, termination conditions, boundary conditions and other system configurations, as well as the individual fitness values and population fitness values of the two algorithms.
Step 2: Initialize population individuals and randomly generate all individuals within the boundary. Individual and population fitness values were calculated and preserved.
Step 3: The genetic algorithm is used to calculate the population and update the fitness value of particles and the fitness value of the population.
Step 4: Judge whether the fitness values of all individuals become better, and use particle swarm optimization algorithm to optimize the degraded individuals.
Step 5: Judge whether the fitness value optimized by particle swarm optimization becomes better and update the fitness value.
Step 6: Update the population fitness value for the next individual direction determination.
Step 7: Judge whether the termination condition is met. If so, end the calculation; otherwise, return to the third step for loop iteration.

### 3.4. Hybrid Optimization Algorithm Based on Two-Stage Adaptive Adjustment

**Figure 4:**
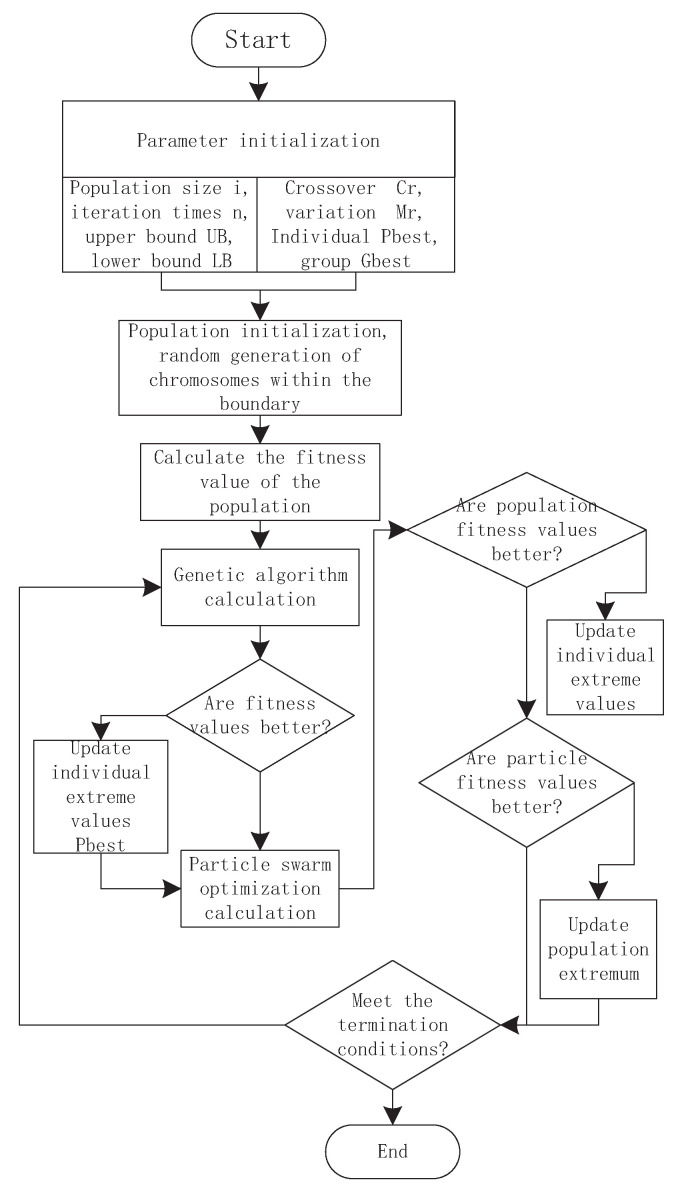
Mine water optimization flow chart based on hybrid algorithm.

In the mixed calculation process of mine water scheduling, the overall defects of the genetic algorithm and particle swarm optimization algorithm have been complementary, but for solving specific cases, the internal details need to be optimized and adjusted. The optimization process is shown in Figure 4. Therefore, this section uses the scheduling process of mine water in each stage and the optimization process of the algorithm to adjust the hybrid algorithm. The first stage is the optimization process of the genetic algorithm. The main factors affecting the performance of genetic algorithm are crossover probability and mutation probability. Crossover probability affects the overall evolutionary process of the algorithm. Through crossover changes between different chromosomes, more excellent individuals appear, while variation mainly plays an auxiliary role. When the algorithm falls into the local optimal solution, mutation operation can make the algorithm jump out of the constraint. Then, it is the second stage, the optimization process of particle swarm optimization algorithm. According to the characteristics of the algorithm, the performance of the algorithm is mainly related to the inertia weight ω; the larger the weight, the stronger the global search ability, the smaller the weight, the stronger the local search ability. To sum up, how to adjust the influence factors of genetic algorithm in the first stage, namely crossover probability and genetic probability, and the influence factors of particle swarm optimization in the second stage are very important.

In the first stage of optimization, the traditional genetic algorithm is fixed for crossover probability and mutation probability, that is, all the particles evolve at a fixed rate, which is easy to cause problems such as oscillation in the optimization process and slow convergence speed. Therefore, in order to solve the above problems and adjust the evolution direction of each particle in the iteration process, based on the analysis of status of individuals in the population to decide the crossover probability and mutation probability of particles, achieve the goal of adaptive evolution of individual particles and improve the optimization performance of the genetic algorithm, which can quickly achieve optimal dispatch of mine water.

Firstly, the fitness value of each particle is determined. In order to fit the characteristics of genetic algorithm and particle swarm optimization algorithm, the reciprocal of the objective function is adopted as the fitness function, and the minimum fitness value is taken as the optimization goal. Namely, the fitness function as Equation (21):

(21) $$fit=\frac{1}{\sum_{i=1}^{M}\sum_{j=1}^{N}\left(\omega_{1}\frac{\left(Y_{ij}+E_{ij}+\delta \right)\times Q_{ij}}{Z\times S}+\omega_{2}\frac{Q_{ij}\times T}{t_{i}\times Z}\right)}.$$

Then, the optimal fitness value $f_{\min}$ and the average fitness value $f_{avg}$ of the population were obtained as Equations (22) and (23):

(22) $$f_{\mathrm{avg}\;}=\cdot \frac{1}{M}\sum_{i=1}^{M}fit$$

(23) $$f_{\min}=\mathrm{argmin}\left\{fit\left(X_{i}\left(1\right)\right),fit\left(X_{i}\left(2\right)\right),fit\left(X_{i}\left(3\right)\right),\ldots ,fit\left(X_{i}\left(k\right)\right)\right\}$$

Through the study of genetic algorithm, it is found that the particle crossover probability between 0.6 and 0.9 is the most suitable [62]. When the fitness value of particles is higher than the average value, the crossover probability of particles is randomly selected between 0.6 and 0.9, and excellent particles are reserved as far as possible. When the fitness value of particles is lower than the average value, the crossover probability of particles is 0.9, that is, the particles with poor mass will be crossed with the maximum probability.

In this paper, the crossover probability of particles will be adjusted according to the individual’s current fitness value in the process of iteration, namely the formula is as Equation (24):

(24) $$f_{i}=\left\{\begin{array}{cc} P_{c1}-\frac{\left(P_{c1}-P_{c2}\right)\left(f_{\mathrm{ave}\;}-f^{\prime }\right)}{f_{\mathrm{avg}\;}-f_{\min}} & f^{\prime }<f_{\mathrm{ave}\;}\\ P_{c1} & f^{\prime }>f_{\mathrm{ave}\;} \end{array}\right.$$

where $P_{c}$ represents the crossover probability of the current particle, $P_{c1}$ represents the maximum crossover probability, which is 0.9; $P_{c2}$ represents the minimum value of crossover probability, which is 0.6; $f^{\prime }$ indicates the fitness value of the current particle; $f_{\min}$ and $f_{\mathrm{avg}}$ represent the minimum fitness value and the average fitness value of the particle population in this iteration, respectively. The unique mutation operation makes genetic algorithm jump out of the problem of local optimum. Adaptive adjustment of particle mutation probability makes genetic algorithm more diverse in the later iteration. The variation probability of particles is more suitable between 0.01 and 0.1 [63]. When the fitness value of the particle is better, the mutation probability of the particle is increased. When the fitness value of particles is poor, the traversal probability of particles is reduced, and the crossover and optimization of particles are emphasized. The formula of mutation operation is Equation (25):

(25) $$P_{m}=\left\{\begin{array}{cc} P_{m1}-\frac{\left(P_{m1}-P_{m2}\right)\left(f_{\mathrm{ave}\;}-f^{\prime }\right)}{f_{\mathrm{ave}\;}-f_{\min\;}} & f^{\prime }\leq f_{\mathrm{avg}\;}\\ P_{m1} & f^{\prime }>f_{\mathrm{avg}\;} \end{array}\right.$$

where $P_{m}$ represents the mutation probability of the current particle; $P_{m1}$ represents the maximum variation probability, which is 0.1; $P_{m2}$ represents the minimum variation probability, which is 0.6; $f^{\prime }$ indicates the fitness value of the current particle; $f_{\min}$ and $f_{\mathrm{avg}}$ represent the minimum fitness value and the average fitness value of the particle population in this iteration, respectively.

When the fitness value of ga particles becomes worse in the iteration process, particle swarm optimization algorithm will replace genetic algorithm for optimization. In order to keep the same adaptive adjustment ability of particle swarm optimization (PSO), this paper will improve the inertia weight. The inertia weight is usually in the range of 0.4–0.95, and W directly affects the particle optimization speed. Referring to the fitness value of the current particle, when the fitness value of the particle is better, the moving speed of the particle should be appropriately reduced. For particles with larger fitness values, they evolve toward the optimal value at maximum speed. Therefore, relevant parameters are set in this paper to make the inertia weight as Equation (26):

(26) $$\omega =\left\{\begin{array}{cc} \omega 1-\frac{\left(\omega 1-\omega 2\right)\left(f_{\mathrm{ave}\;}-f^{\prime }\right)}{f_{\mathrm{ave}\;}-f_{\min}} & f^{\prime }\leq f_{\mathrm{avg}\;}\\ \omega 1 & f^{\prime }>f_{\mathrm{avg}\;} \end{array}\right.$$

$\omega_{1}$ indicates the maximum inertia weight, set to 0.95, $\omega_{2}$ indicates the minimum inertia weight, set to 0.4. In the initial stage of the algorithm, larger *f* and larger ω are more conducive to global search; on the contrary, in the late running state of convergence, smaller *f* and smaller ω are more conducive to local search.

In conclusion, this section of important parameters in the GAPSO algorithm are optimized and improved. The original fixed parameters improvement for change with the particle population increased the variability of the algorithm, in theory, to improve the speed of searching optimization and optimization precision of the particle, with the improved algorithm called two-stage adaptive genetic particle swarm optimization (TSA-GAPO).

## 4. Case Analysis and Discussion

In order to verify the feasibility of the TSA-GAPSO algorithm in dealing with the mine water optimal scheduling problem, this chapter uses the TSA-GAPSO algorithm to calculate the economic reuse mathematical model of mine water and compares it with the PSO algorithm, GA algorithm and GAPSO algorithm to observe the convergence state of the four optimization algorithms. Then, water in 2015 Dahaize mining areas is redistributed and scheduled, and its reuse cost and reuse rate are analyzed, so as to verify the superiority of the TSA-GAPSO algorithm for optimal allocation of mine water.

### 4.1. Algorithm Simulation Analyses

**Figure 5:**
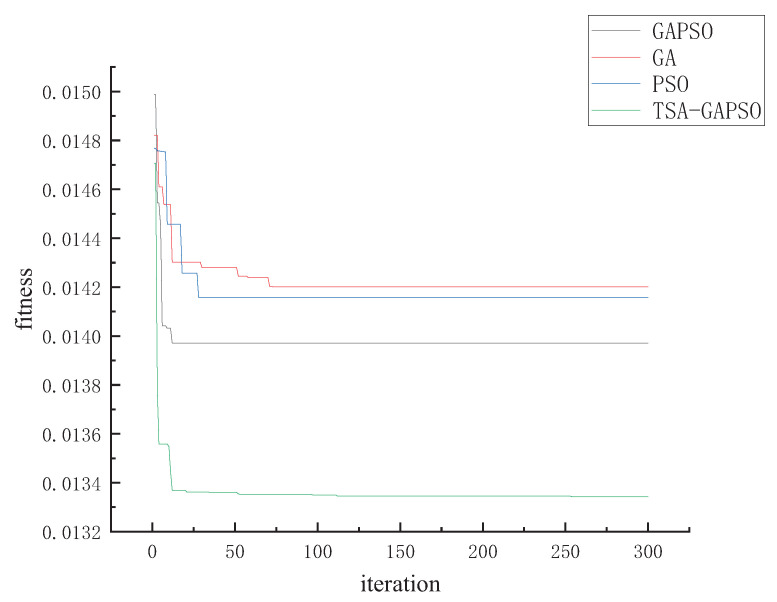
Comparison diagram of algorithm optimization.

In this section, the TSA-GAPSO algorithm is used to simulate the scheduling model of mine water, so as to verify the feasibility of the algorithm in optimal distribution of mine water. The iteration of the four algorithms is shown in Figure 5, and the statistical index data of algorithm optimization for the mine water scheduling model is shown in Table 1. Python, as an interpreted high-level programming language, has penetrated into hot fields such as big data and artificial intelligence. Based on the advantages of algorithmic editing, the testing process was completed on Python software. The number of particle swarm in this paper is 100, and the maximum iteration number is 300. Each particle contains four features. Finally, the minimum fitness value, average value and iteration time of the algorithm after iteration is taken as evaluation criteria.

As can be seen from the figure, TSA-GAPSO has a lower fitness value than the single GA algorithm, PSO algorithm and mixed GAPSO algorithm in terms of convergence accuracy, that is, it has a better convergence effect. In terms of convergence speed, it can be seen from the figure that TSA-GAPSO, GAPSO and PSO algorithms reach the optimum at about 12 times, while GA algorithm reaches the optimum at about 75 times. Therefore, the first three algorithms had better convergence accuracy.

In terms of convergence speed, GA algorithm is optimal at about 20 iterations, PSO is optimal at about 30 iterations and GAPSO is optimal at about 15 iterations. Compared with GA algorithm and PSO algorithm, GAPSO had better convergence accuracy. Therefore, in terms of comprehensive evaluation, TSA-GAPSO has relatively good convergence state. This paper compares the algorithms mentioned in References [34,35,36] and finds that they are similar to Reference 30 and better than the convergence speed of the algorithms in the other two papers. In order to verify the stability of the TSA-GAPSO algorithm and the superiority of the TSA-GAPSO algorithm in the mine water scheduling model, this paper has performed 100 simulation experiments. The maximum value, minimum value and the average value of each algorithm are counted, and then the differences of each algorithm are analyzed.

As can be seen from Table 1, the overall effect is similar to that in Figure 5. Among the four algorithms, TSA-GAPSO has the best optimization effect, reaching 1.35E-02. GAPSO followed with 1.39E-02. The optimization degree of single GA algorithm is the worst. It is proved that the hybrid algorithm is better than the single algorithm in mine water optimal scheduling. As can be seen from the minimum value, the minimum value of PSO is the smallest, 9.11E-03, followed by TSA-GAPSO, 1.32E-02. However, as can be seen from the different value, the fluctuation range of PSO is large, about 6 times that of TSA-GAPSO, and the relative stability is poor. Through the above analysis, TSA-GAPSO has a good comprehensive evaluation effect and has a good optimization performance in mine water optimization scheduling.

### 4.2. Case Verification Analyses

Next, the mine water in 2015 in the Dahaize mining area will be redistributed to compare the reuse cost of the four algorithms in mine water scheduling and verify the practicality and superiority of the hybrid improved algorithm.

**Figure 6:**
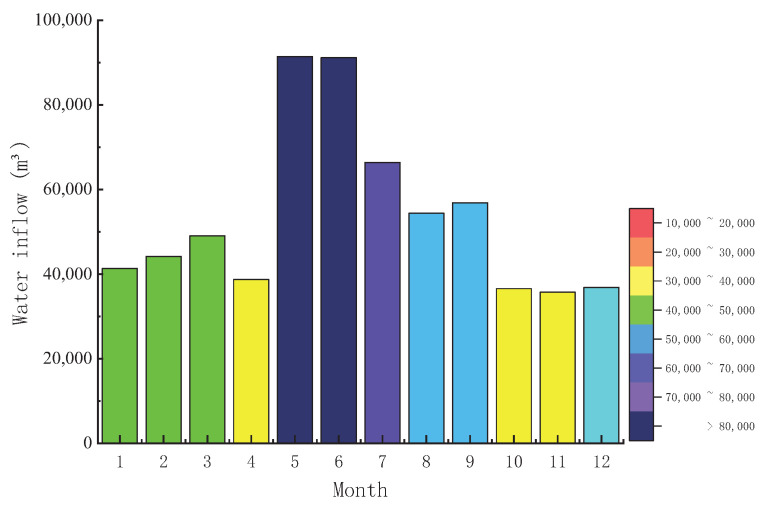
Water inflow in Dahaize coal mine.

It can be learned from the first chapter that there are four water pools of mine water reuse: underground clean pool, middle pool, high pool and reuse pool. Water has 14 points, mainly for downhole hydraulic support, grouting in the use of water, cooling water, underground water underground mine fire and dust removal, etc., five water points, and ground fire water, dust, coal in the use of water, heat exchanger station water, cooling water, boiler water, green water, drinking water and other water with nine points. According to the survey of the mining area, the water inflow in the mining area far exceeds the water consumption, so the dispatching and distribution in this paper does not need external water supply, and the monthly water inflow is shown in Figure 6.

**Figure 7:**
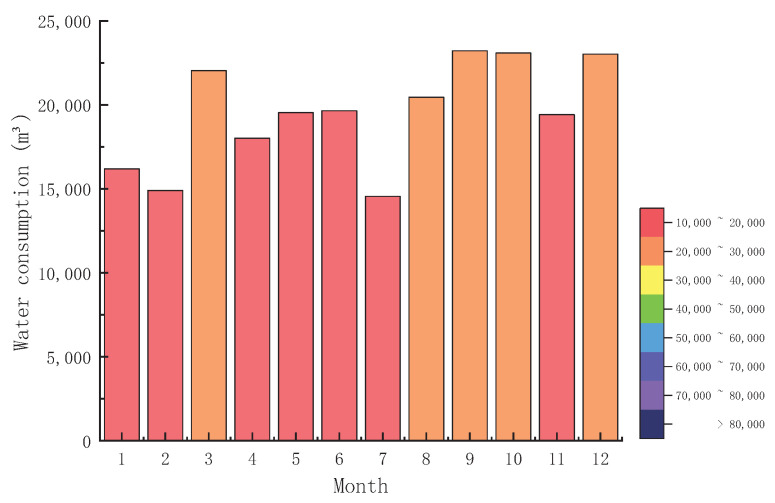
Bar chart of water consumption in mining area.

According to the investigation of water consumption mechanism and water consumption in the mining area, it can be found that the dispatching mode adopted in the mining area is the nearest principle, that is, when the water point is used, the nearest water supply point will supply, which will inevitably lead to the accumulation of water supply and unreasonable dispatching. Therefore, this paper will use the optimization algorithm to redistribute mine water and compare it with the nearest distribution method to verify the superiority of the optimization algorithm in the reuse efficiency and cost. The annual water consumption of the mining area is shown in Figure 7.

**Figure 8:**
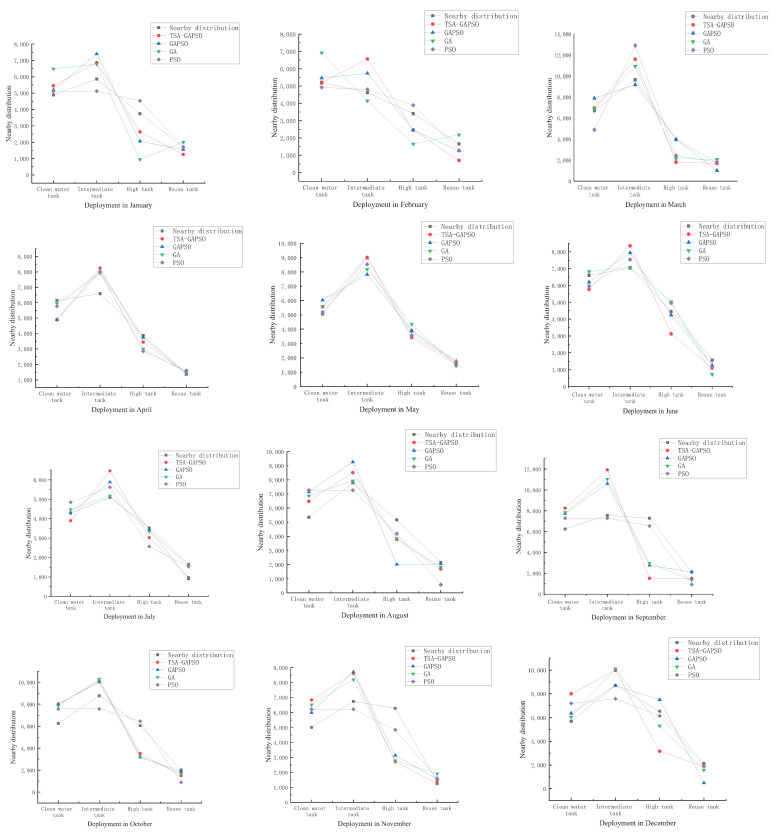
Mine water and moon dispatching situation.

The distribution map of water consumption in the mining area of wetland was obtained through investigation. As shown in Figure 8, the monthly water consumption in the mining area is between 15,000 and 20,000. The amount of water used fluctuates and adjusts according to the actual situation. Through the analysis and calculation of water consumption, it can be known that the recycling rate of mine water cannot reach 100%. As a result, surplus mine water will be discharged into the environment or transported to the nearest water treatment plant upon completion of treatment after meeting the mine need as far as possible.

In the normal operation process, the concept of green mining should be followed, so all mine water needs to be treated. Therefore, mine water scheduling belongs to reuse in the process of treatment and has relatively little influence on the process of treatment. Optimal scheduling mainly refers to the selection of optimal scheduling schemes by using algorithms.

It can be seen from the scheduling situation of the group of graphs that the algorithm redistributes the scheduling water amount of mine water. In most cases, the intermediate pool has the largest amount of dispatching, because the water quality of the intermediate pool meets the surface production water in most mining areas and the cost is relatively low. Due to the limitation of water quality, some mine water can only be regulated by fixed pools, for example, drinking water can only be regulated by multiplexed pools, so the number of multiplexed pools also exist all the time. There is also a relatively large amount of dispatching water in underground clean water pools, because it takes a lot of dispatching time and economic cost to dispatch underground water resources to the surface. Therefore, water from underground clean water pools is generally used to allocate underground water.

The amount of each pool is scheduled at the overall optimal recycling cost under the constraints of the restrictive conditions. The following compares the scheduling cost to analyze the optimization cost of various algorithms and the operating efficiency of the processing system.

It can be seen from Table 2 that the recycling cost of mine water dispatching is the highest in the fourth quarter, because the recycling amount of mine water increases in this quarter. On the whole, the reuse cost of mine water varies slightly according to different optimization methods. TSA-GAPSO algorithm has the highest cost reduction rate, reaching 9.09%. The scheduling recycle cost based on the GA algorithm is the lowest but also reaches 6.67%. As can be seen from the table, with the reduction in cost, the running time of the mine water treatment system is reduced, that is, the efficiency of optimal scheduling will also be improved accordingly. On the basis of nearby scheduling, optimization efficiency based on TSA-GAPSO is improved by 5.81%, and optimization efficiency based on GAPSO, GA and PSO is improved by 3.95%, 2.99% and 3.85%, respectively. To sum up, the TSA-GAPSO based mine water optimal scheduling algorithm proposed in this paper has good practicability and superiority.

## 5. Conclusions

Based on the analysis of the operation characteristics of the treatment system and the current situation of water consumption in the mining area, this paper constructs the water demand model of the mining area. The economic reuse objective function is designed according to the demand of mining area. In order to calculate the objective function, according to the characteristics of the GA algorithm and PSO algorithm, this paper proposes a GAPSO algorithm which uses PSO algorithm to assist optimization on the basis of the GA algorithm mechanism and improves it. In addition, the utilization function of mine water is used to compare the allocation of mine water and the performance of the algorithm before and after improvement to verify its effectiveness. The convergence results show that the optimal scheduling has higher cost and efficiency than the traditional scheduling, and the hybrid improved algorithm can obtain more mine water allocation schemes in less iterations than the basic algorithm. The theory presented in this paper can provide theoretical support for mine water and other scheduling problems. However, the main purpose of this paper is the reuse cost of mine water. We will further improve the reuse mechanism and effect of mine water, such as the introduction of an evaluation mechanism, to make our experiment more real. Finally, we will study how to experience the reserved mine water scheduling resources and plan the scheduling scheme according to the preset scheduling path.

## Figures and Tables

**Table 1:** The algorithm statistics index data of mine water dispatching model.

Algorithm	Max	Min3	D-Value	Average
TSA-GAPSO	1.42E-02	1.32E-02	1.00E-02	1.35E-02
GAPSO	1.44E-02	1.35E-02	9.06E-03	1.39E-02
GA	1.68E-02	1.54E-02	1.42E-02	1.59E-02
PSO	1.53E-02	9.11E-03	6.20E-03	1.47E-02

**Table 2:** The algorithm statistics index data of mine water dispatching model.

Scheduling Condition	Dispatching Recycling Cost (Ten Thousand CNY)				Total	Cost Reduction Rate (%)	Running Time (h)	Efficiency Improvement (%)
	The First Quarter	The Second Quarter	The Third Quarter	The Fourth Quarter				
Schedule to the nearest	6.72	7.04	7.76	8.51	30.04	-	35,040.00	-
TSA-GAPSO	6.03	6.70	6.72	7.86	27.31	9.09	32,879.85	5.81
GAPSO	6.20	6.97	6.93	7.57	27.67	7.89	33,599.04	3.95
GA	6.51	6.85	7.00	7.83	28.20	6.13	339,59.91	2.99
PSO	6.65	6.80	6.57	8.02	28.04	6.67	33,637.83	3.85

## Data Availability

The data that support the findings of this study are available from the corresponding author upon reasonable request.
